# Biofilms formed by *Candida albicans* bloodstream isolates display phenotypic and transcriptional heterogeneity that are associated with resistance and pathogenicity

**DOI:** 10.1186/1471-2180-14-182

**Published:** 2014-07-05

**Authors:** Leighann Sherry, Ranjith Rajendran, David F Lappin, Elisa Borghi, Federica Perdoni, Monica Falleni, Delfina Tosi, Karen Smith, Craig Williams, Brian Jones, Chris J Nile, Gordon Ramage

**Affiliations:** 1 Infection and Immunity Research Group, Glasgow Dental School, School of Medicine, College of Medical, Veterinary and Life Sciences, University of Glasgow, 378 Sauchiehall Street, Glasgow G2 3JZ, UK; 2 Laboratory of Microbiology, Department of Health Sciences, Università degli Studi di Milano, Milan, Italy; 3 Division of Human Pathology, Department of Health Sciences, Università degli Studi di Milano, Milan, Italy; 4 Institute of Healthcare Associated Infection, School of Health, Nursing and Midwifery, University of the West of Scotland, Paisley, UK; 5 Microbiology Department, Glasgow Royal Infirmary, Glasgow, UK

**Keywords:** *Candida albicans*, Biofilm, Candidaemia, Antifungal

## Abstract

**Background:**

*Candida albicans* infections have become increasingly recognised as being biofilm related. Recent studies have shown that there is a relationship between biofilm formation and poor clinical outcomes in patients infected with biofilm proficient strains. Here we have investigated a panel of clinical isolates in an attempt to evaluate their phenotypic and transcriptional properties in an attempt to differentiate and define levels of biofilm formation.

**Results:**

Biofilm formation was shown to be heterogeneous; with isolates being defined as either high or low biofilm formers (LBF and HBF) based on different biomass quantification. These categories could also be differentiated using a cell surface hydrophobicity assay with 24 h biofilms. HBF isolates were more resistance to amphotericin B (AMB) treatment than LBF, but not voriconazole (VRZ). In a *Galleria mellonella* model of infection HBF mortality was significantly increased in comparison to LBF. Histological analysis of the HBF showed hyphal elements intertwined indicative of the biofilm phenotype. Transcriptional analysis of 23 genes implicated in biofilm formation showed no significant differential expression profiles between LBF and HBF, except for *Cdr1* at 4 and 24 h. Cluster analysis showed similar patterns of expression for different functional classes of genes, though correlation analysis of the 4 h biofilms with overall biomass at 24 h showed that 7 genes were correlated with high levels of biofilm, including *Als3*, *Eap1*, *Cph1*, *Sap5*, *Plb1*, *Cdr1* and *Zap1.*

**Conclusions:**

Our findings show that biofilm formation is variable amongst *C. albicans* isolates, and categorising isolates depending on this can be used to predict how pathogenic the isolate will behave clinically. We have shown that looking at individual genes in less informative than looking at multiple genes when trying to categorise isolates at LBF or HBF. These findings are important when developing biofilm-specific diagnostics as these could be used to predict how best to treat patients infected with *C. albicans*. Further studies are required to evaluate this clinically.

## Background

Bloodstream infections (BSI) caused by *Candida* species remain a frequent cause of morbidity and mortality, particularly within the immunocompromised population [1,2]. Overall, *Candida* species have been identified as the most common fungal pathogen found in bloodstream infections in the United States, and are the fourth most common organism responsible for all BSI, and are the third most common within the intensive care unit (ICU) [2]. Candidaemia is often associated with the ability of *Candida* to adhere to and form biofilms on indwelling medical devices, such as central venous catheters (CVC) and prosthesis [3,4]. Biofilms are a population of microorganisms attached to one another and/or a surface, surrounded by an extracellular matrix (ECM) [5].

A defining feature of biofilms is their resistance to antimicrobial therapy, with higher drug concentrations required to kill biofilms and their dispersed cells when compared to equivalent free-floating planktonic cells [5-7]. Another feature of *C. albicans* biofilms is their enhanced pathogenicity. For example, cells detaching from biofilms have been shown to be more cytotoxic than their planktonic counterparts and significantly increase mortality within a murine model of infection [7]. These observations have been demonstrated clinically, where a significant association was observed between *C. albicans* biofilm formation and mortality rates in candidaemia patients [8].

Whilst there is growing evidence of the importance of *Candida* biofilms in clinical medicine, not all clinical isolates are able to form biofilms. There is therefore a fundamental gap in understanding exactly what drives biofilm formation and its clinical implications. Establishing methods to differentiate these isolates is challenging, as many studies rely on either metabolic assays or biomass, and these frequently use a variety of different substrates and media [9-12]. Therefore, comparison between these studies is not possible, and further interpretation of the data to improve clinical management both for diagnostics and antifungal therapy is limited. The purpose of this study was therefore to investigate and characterise biofilm formation by clinical isolates of *C. albicans* using standard methodologies and subsequently analyse biofilm subsets phenotypically and transcriptionally. Here we report that *C. albicans* clinical isolates form biofilms that are heterogeneous, and this is associated with altered antifungal drug sensitivity and pathogenic potential.

## Results

### *Candida albicans* clinical isolates exhibit heterogeneous biofilm formation

**Figure 1:**
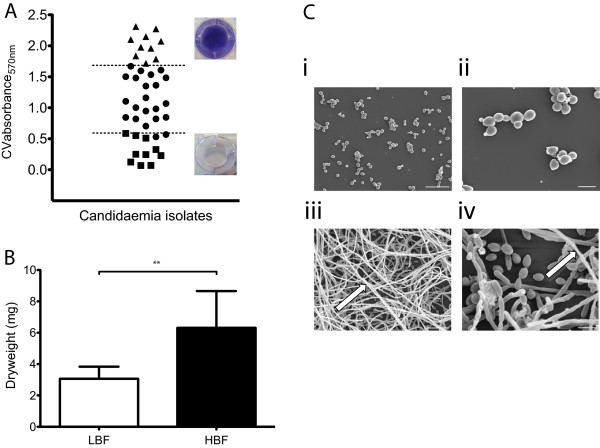
***Candida albicans *clinical isolates vary in their ability to form biofilms.** Forty-two *C. albicans* bloodstream isolates were used to evaluate biofilm formation of strains derived from a clinical setting. **(A)** Standardised *C. albicans* (1 × 10^6^ cells/mL) in RPMI-1640 were grown in flat-bottomed 96 well microtitre plates for 24 h at 37°C. Mature biofilms were carefully washed with PBS, allowed to air dry and biomass quantified by staining with 0.05% w/v crystal violet solution. The biofilms were washed and destained with 100% ethanol. Biomass was quantified spectrophotometrically by reading absorbance at 570 nm in a microtitre plate reader (FluoStar Omega, BMG Labtech). Eight replicates were used for each isolate and was carried out on two separate occasions, with the mean of each represented. *C. albicans* LBF (square), HBF (triangle) and IBF (circle) were defined by the upper and lower quartiles, as shown by crystal violet stained biofilms. **(B)** Three *C. albicans* LBF and HBF were standardised (1 × 10^6^ cells/mL) in RPMI-1640 and grown in 12 well plates for 24 h at 37°C. Biofilms were washed with PBS, biomass scraped and passed through 0.22 μm filters before the filters containing the biofilms were dried at 37°C for 24 h. Biofilm dry weight was then measured for LBF and HBF, in triplicate on three separate occasions. Data represents mean ± SD with significance **p < 0.005. **(C)** One *C. albicans* LBF **(i, ii)** and HBF **(iii, iv)** were grown on Thermanox™ coverslips for 24 h at 37°C. Biofilms were then processed and viewed on a JEOL JSM-6400 scanning electron microscope and images assembled using Photoshop software. Note the lack of biomass and hyphal cells in LBF. Scale bars represent 20 μm and 5 μm for 1000× **(i, iii)** and 3000× **(ii, iv)** magnifications, respectively.

*C. albicans* bloodstream isolates displayed heterogeneity with respect to their biofilm biomass when grown in RPMI (Figure 1A). RPMI was shown to support the optimal growth of *C. albicans* over 24, 48 and 72 h (Additional file 1: Figure S1). Isolates were categorised as low biofilm formers (LBF) or high biofilm formers (HBF) if their biomass absorbance were less than the first quartile (Q_1_ OD_570_ = 0.565) or greater than the third quartile (Q_3_ OD_570_ = 1.682), respectively. Those isolates in between the first and third quartile (Q1-Q3) were defined as intermediate biofilm formers. When HBF were stained with crystal violet (cv), the extent of the biofilm formation was observed macroscopically, where the bottom of the well was clearly covered with cellular biomass (Figure 1A). In contrast, minimal staining was retained on isolates classed as LBF, as demonstrated by the well remaining almost colourless. We analysed a subset of isolates from the LBF and HBF group (n = 3) using dry weight measurements and confirmed our previous observations that biofilm biomass was significantly greater in isolates termed HBF (p = 0.0023) (Figure 1B). These differences are clearly evident when viewed under a SEM at low (Figure 1C [i, iii]) and high magnification (Figure 1C [ii, iv]). LBF isolates were characterised by a predominance of yeast cells and lack of hyphal cells (Figure 1C i, ii). In contrast, *C. albicans* HBF were highly filamentous with a multi-dimensional structure with very few yeast cells (Figure 1C iii, iv).

### Biofilm phenotype is affected by cell surface hydrophobicity (CSH)

**Figure 2:**
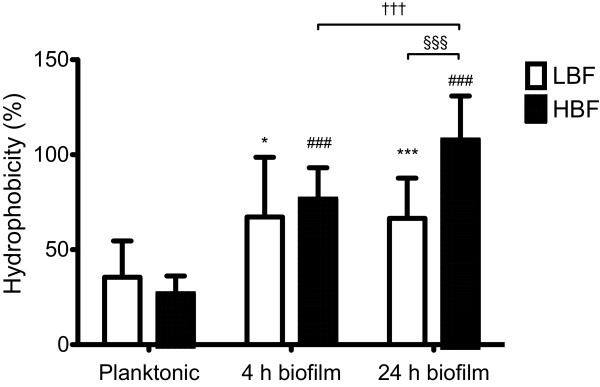
**Cell surface hydrophobicity impacts biofilm phenotype.** Ten *C. albicans* LBF and HBF were standardised (1 × 10^6^ cells/mL) in RPMI-1640 and grown in 75 cm^2^ flasks for 4 and 24 h. Biofilms were washed with PBS, biomass scraped in to YPD media and standardised to OD_590nm_ 1.0 before xylene was added to each sample. Planktonic cells were also standardised to OD_590nm_ 1.0. Samples were allowed to separate into two phases and the OD_590nm_ of the lower aqueous layer was measured **(i)**. A visual representation hydrophobicity is shown for planktonic LBF **(ii)** and HBF **(iii)**, 4 h biofilms LBF **(iv)** and HBF **(v)** and 24 h biofilms LBF **(vi)** and HBF **(vii)**. Note the cloudy upper layer denoted by arrows showing hydrophobic cells. Ten isolates from each group were measured on two separate occasions. Data represented mean ± SD. Significant differences between LBF and HBF were observed when 4 and 24 h biofilms were compared to their planktonic counterparts (*p < 0.05, ***p < 0.0001,^###^*p < 0.0001*). Furthermore, significant differences were found between 4 and 24 h in HBF (^†††^*p < 0.0001*) and between LBF and HBF at 24 h (^§§§^*p < 0.0001*).

The CSH of LBF and HBF isolates was quantified to determine whether it played a role in biofilm forming ability [13]. Figure 2 illustrates that the hydrophobicity of an isolate significantly alters its ability to form a biofilm. For LBF the CSH increased by 32% and 31% in 4 h (p < 0.05) and 24 h (p < 0.0005) biofilms, respectively, when compared to planktonic cells. This trend was also observed in isolates with HBF where CSH increased by 50% in 4 h (p < 0.0001) biofilms and 81% in 24 h (p < 0.0001) biofilms, when compared with planktonic counterparts. Furthermore, a significant increase in CSH was found in isolates with HBF between early (4 h) and mature (24 h) phases of biofilm development, where hydrophobicity increased by 31% (p < 0.001). When the hydrophobicity of LBF and HBF was compared, CSH was significantly increased by 41% in HBF isolates at 24 h (p < 0.0001); however, no significant difference was observed between isolates with LBF and HBF in planktonic cells and 4 h biofilms.

### Amphotericin B activity is impacted by biofilm phenotype

**Figure 3:**
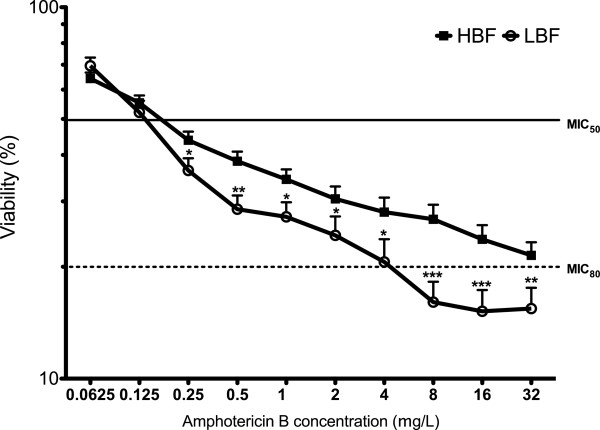
**Amphotericin B sensitivity is significantly impacted by biofilm formation.** Ten isolates with LBF and HBF were standardised to 1 × 10^6^ cells/mL in RPMI-1640 and grown as biofilms in flat-bottomed 96 well microtitre plates for 24 h. Biofilms were washed with PBS before treated with 2 fold serial dilutions of amphotericin B for 24 h. Biofilms were washed and metabolic activity measured using the XTT metabolic assay with absorbance read at 492 nm. Each isolate was tested in duplicate, on three separate occasions with data represented by mean ± SEM. *p < 0.05, **p < 0.01, ***p < 0.001.

Sessile antifungal testing was performed on *C. albicans* isolates with LBF and HBF to determine if one group were more susceptible to VRZ or AMB treatment. VRZ was ineffective against all biofilms tested, showing no difference in activity against LBF and HBF (data not shown). However, a dose-dependent effect was evident in all isolates tested with AMB (Figure 3). Moreover, a significant difference was observed between LBF and HBF treated with 0.25 – 32 mg/L AMB (p < 0.05). LBF and HBF isolates both had a MIC_50_ of 0.25 mg/L AMB, yet isolates with LBF were significantly less viable than those with HBF at this concentration (p = 0.0307). In addition, LBF isolates achieved an ~80% kill at 4 mg/L, whereas HBF required 32 mg/L to reach the same kill. No significant differences were observed in the growth rates of either set of LBF and HBF isolates (data not shown).

### *In vivo* pathogenicity is affected by biofilm phenotype

**Figure 4:**
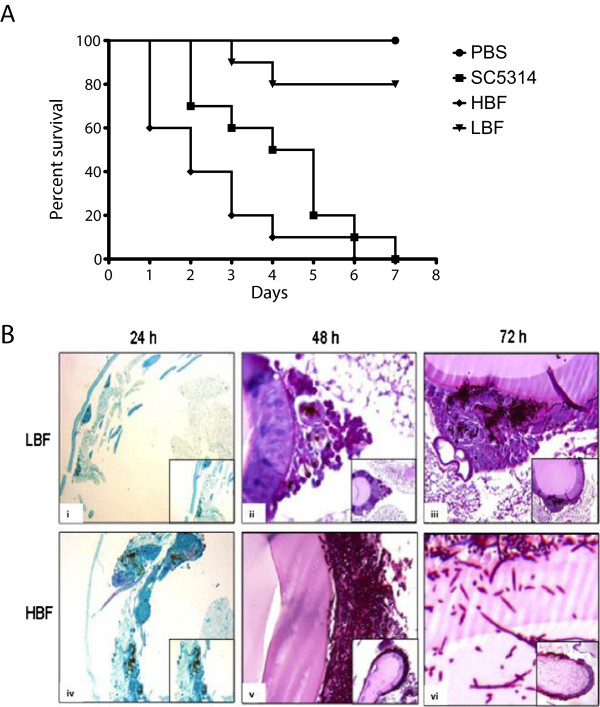
***C. albicans *HBF have a significant impact on morbidity and mortality rate *in vivo*.** Larvae of *G. mellonella* were infected with *C. albicans* LBF or HBF at 1 × 10^5^ CFU/larva and monitored over a period of 7 days **(A)**. Kaplan-Meier plots of *G. mellonella* survival after injection of *C. albicans* demonstrated a strain dependant variation in pathogenicity *in vivo*. Groups of HBF and LBF clinical isolates were compared to each other and to the SC5314 type strain. The HBF isolates resulted in higher killing rate compared to LBF and SC5314. In contrast, LBF isolates exhibit a slower rate of kill and 100% mortality did not occur within 7 days. PBS injected larvae were included as a negative control. **(B) I**nfected larvae were formalin fixed and sectioned for histology analysis. At 24 h, LBF infected larvae **(i)** had several melanisation spots and nodules were present mainly under the cuticle and in the peripheral fat body (Feulgen staining, 20× original magnification (o.m.); inset: 4× o.m.), whereas HBF infected larvae **(iv)** had larger nodules with a greater melanin deposition characterised by the recruitment in the external layers of a huge number of haemocytes (20× o.m.; inset: 10× o.m). At 48 h, LBF **(ii)** small nodules containing both yeast and some hyphae were observed deeper in the larval tissues, sometimes reaching the external part of the gut wall (PAS staining, 20× o.m.; inset: 10× o.m), with HBF **(v)** having elongated hyphae targeting the intestinal walls (PAS staining, 40× o.m.; inset: 10× o.m.) At 72 h, LBF **(iii)** showed segmental invasion of the gut walls (PAS staining, 20× o.m.; inset: 10× o.m.) however, HBF **(vi)** displayed hyphae endoluminal invasion after breaching the intestinal wall (PAS staining, 40× o.m.; inset: 10× o.m.) with few yeast cells.

We next analysed the impact of the isolates ability to form biofilms based upon the severity of infection using a previously described *G. mellonella* model. The average rate of killing by three HBF, three LBF and a reference strain (SC5314) of *C. albicans* were calculated to plot a survival curve. Survival data showed a significant difference in the killing of larvae between HBF and LBF (p < 0.0001 [Figure 4A]). After 2 and 6 days, respectively, >50% and 100% larval death was recorded for HBF isolates, whereas larvae infected with LBF only achieved 20% killing after 7 days challenge. The reference strain SC5314 achieved 50% and 100% larval death by day 4 and 7, respectively. Similar kill rates to that of HBF were observed in the type strain however, when compared to LBF there was a significant difference in larval mortality (p = 0.0005).

Host-pathogen interactions in this model were then investigated by microscopically observing the morphology of the infected larvae at 24, 48 and 72 h post-infection with *C. albicans* HBF and LBF (Figure 4B). At 24 h in both the LBF (Figure 4Bi) and HBF (Figure 4Biv), the nodule formation and melanin deposition were mainly observed under the cuticle and in the fat body, with mild to strong melanisation observed in the centre of the nodules, together with the presence of yeast cells and/or hyphae. The LBF nodules were smaller in dimension and dispersed mainly in the subcuticle area (Figure 4Bi), whereas the HBF nodules had a stronger melanisation with the tendency to converge in large aggregates, and were localised more deeply within the fat body (Figure 4Biv). At 48 h, the LBF were confined to the external part of the visceral organs, with a spot-like distribution (Figure 4Bii); whereas the HBF were found to display a pronounced filamentation all around the intestinal wall, with a PAS positive matrix visible surrounding the hyphae (Figure 4Bv). Furthermore at 72 h, there was a substantial invasion of both the gastrointestinal tract and the tracheal system with damaged gut epithelium, where yeast and hyphal cells both observed in the HBF infection (Figure 4Bvi). In contrast, a segmental invasion of the intestinal wall (Figure 4Biii) was observed with LBF infection and the progression of the infection was to a lesser extent than that by the HBF. Table 1 summarises the localisation and characterisation of the nodules with LBF and HBF infected larvae. Changes in the fat body morphology and composition including vacuolisation and haemocyte recruitment, were detected during the course of the infection and were more evident in the HBF.

**Table 1:** **Characteristics and localisation of nodules found in infected *G. mellonella *** **larvae**

	**Nodules**									
	Size	Melanisation	Encapsulation	Confluence	Fungal morphology		Localisation			
					Yeast cells	Hyphae	SC	FB	PI	PT
**LBF**	Small	+	+	-	+++	+*	+	+	++	-
**HBF**	Large	++, +++	+++	+	+	+++**	++	++	+++	+

SC: subcuticle, FB: fat body, PI: paraintestinal, PT: paratracheal.

*short squat hyphae, **long tangled hyphae often embedded in an extracellular matrix.

### Transcriptional heterogeneity is associated with biofilm phenotype

**Figure 5:**
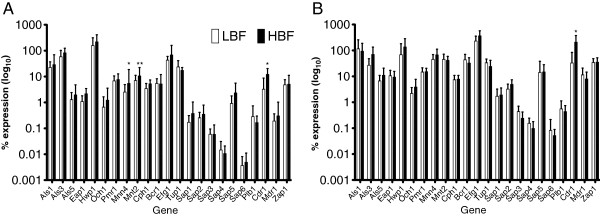
**Genes associated with *C. albicans *biofilm development are up-regulated in HBF.** Ten *C. albicans* isolates with LBF and HBF were standardised to 1 × 10^6^ cells/mL in RPMI-1640 and grown as biofilms in 24 well microtitre plates for 4 **(A)** and 24 h **(B)** at 37°C. Biofilms were washed with PBS and RNA extracted using the TRIzol method, cDNA synthesised and real-time PCR used to measure the expression of genes related to *C. albicans* biofilm formation. Percentage of gene expression is shown as log_10_ mean ± SD, relative to housekeeping gene *ACT1*. All strains were assessed in duplicate and included appropriate no RT and non-template controls. *p < 0.05, **p < 0.005.

*C. albicans* clinical isolates defined as LBF and HBF were further assessed at a transcriptional level and the expression of genes related to biofilm formation was investigated. *ACT1* was used as the housekeeping gene and was shown to be stably expressed throughout all biofilm conditions. Figure 5 illustrates the levels of gene expression of LBF (n = 10) versus HBF (n = 10) at both (A) 4 and (B) 24 h. Overall, the majority of the genes tested followed a trend of up-regulation in HBF compared to LBF. However, statistically significant differences were observed in the glycosylated mannoproteins *MNN4* (p = 0.0313) and *MNT2* (p = 0.0044) at 4 h, where expression was increased by ~2 fold. Furthermore, the resistance gene *CDR1* was significantly increased in HBF by 4- and 6-fold at 4 h (p = 0.0113) and 24 h (p = 0.0239), respectively (Additional file 2: Table S1).

**Figure 6:**
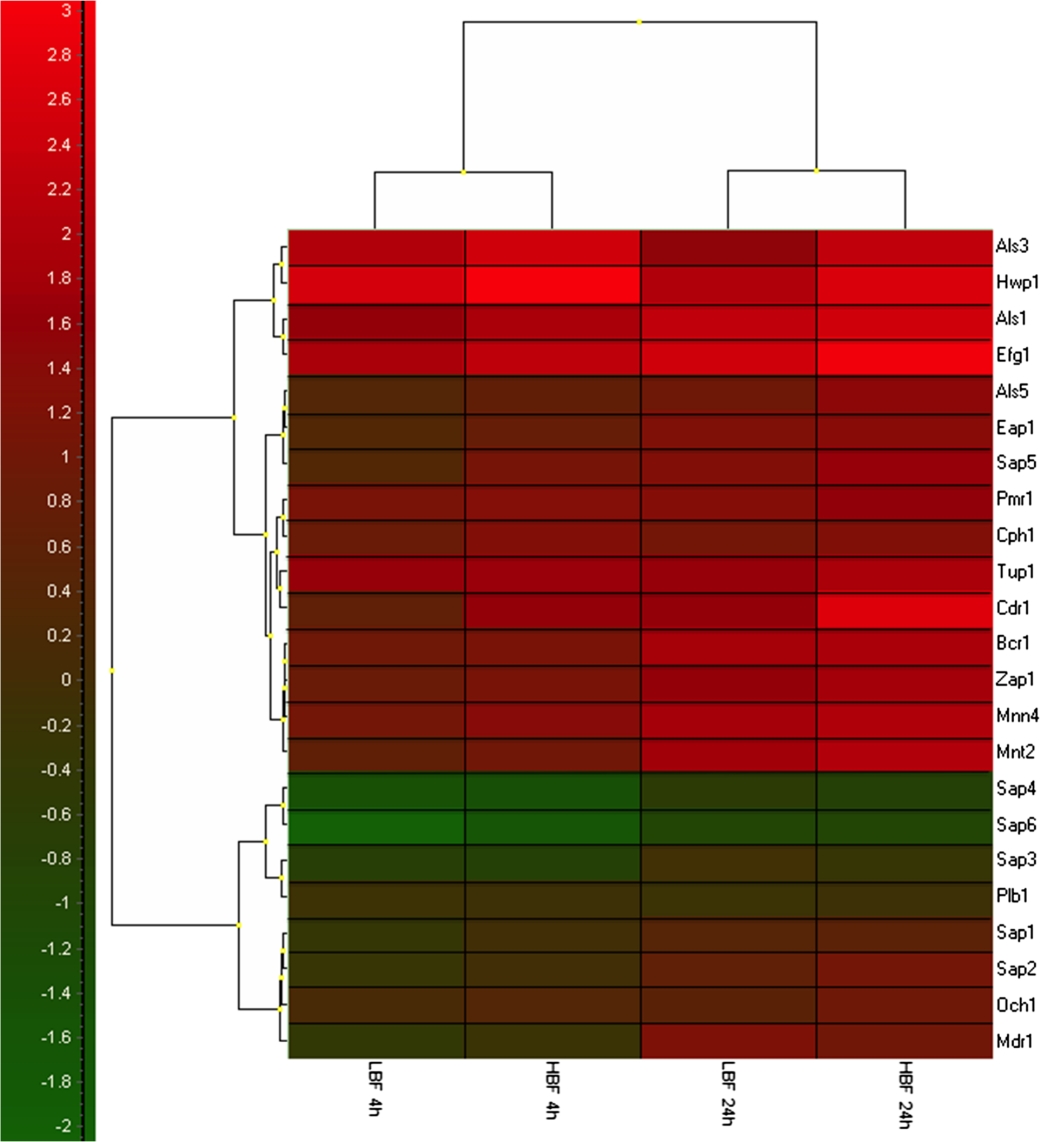
**Clustering analysis identified the transcriptional relationship of biofilm specific genes.** Percentage expression of each gene was also assessed by clustering and heat map analysis using GenEx software. Data was log transformed and mean values were used for heat map construction. Increased expression of genes is shown by red and a decrease is represented by green.

Clustering the expression of 23 selected genes from 5 different functional groups in a heat map showed their relationship with one another and their variable expression in LBF and HBF over time (Figure 6). Here we found the adhesion genes *ALS3* and *HWP1* were closely related and highly expressed, particularly in HBF isolates at 4 and 24 h. Furthermore, genes from different functional groups were closely related to one another irrespective of whether LBF or HBF, such as the proteinase *SAP5* and the adhesion genes *ALS5* and *EAP1*. The remaining *SAP* genes were all closely related to one another, and interestingly the resistance gene *MDR1* and the cell wall mannoprotein *OCH1*. Further analysis of *SAP3* showed an increase in transcription within LBF at 24 h, despite no differences being observed at 4 h. In contrast, *SAP5* expression was consistently high at 4 and 24 h within HBF.

Analysis of Spearman rho coefficients found that out of 23 selected genes, 7 including those related to adhesion (*ALS3, EAP1*), filamentation (*CPH1*), hydrolytic enzymes (*SAP5*, *PLB1*) and resistance (*CDR1 and ZAP1*) showed a significant positive correlation (p < 0.05) with cv biomass data at 4 h (Table 2). Further analysis of the relationship between these seven genes and all the other genes tested presented various correlations (marked bold in Table 2). For example, *PLB1* was significantly correlated with all other genes tested (94.11%) except *ZAP1*, followed by *CPH1* (76.47%)*, SAP5* (76.47%)*, EAP1* (64.71%)*, CDR1* (41.17%), *ALS3* (35.29%) and *ZAP1* (11.76%)*.* Correlation of individual genes with one another at the 4 h time point showed that *PLB1*, *CPH1*, *MNN4* and *HWP1* all correlated with 5 of the 7 key genes defined above. Notably, 24 h gene expression revealed very few significant correlations other than cv. In fact, a significant negative correlation was found between *SAP3* and the biomass data (Rho = −0.465, p = 0.045). Furthermore, *SAP3* was positively correlated with *MNT2* (Rho = 0.468, p = 0.043) and *SAP4* (Rho = 0.460, p = 0.048).

**Table 2:** ***C albicans *** **biomass correlates with biofilm-related gene expression**

**4 h Correlations**^**a**^		***ALS3***	***EAP1***	***CPH1***	***SAP5***	***PLB1***	***CDR1***	***ZAP1***
CV	Rho=	.529^*^	.608^**^	.534^*^	.539^*^	.483^*^	.647^**^	.515^*^
	p =	**.029**	**.010**	**.027**	**.026**	**.050**	**.005**	**.035**
*ALS1*	Rho=	.240	.529^*^	.542^*^	.336	.608^**^	.382	.250
	p =	.353	**.029**	**.025**	.188	**.010**	.130	.333
*ALS3*	Rho=	1.000	.385	.708^**^	.544^*^	.593^*^	.779^**^	.365
	p =		.127	**.001**	**.024**	**.012**	**.000**	.149
*ALS5*	Rho=	.229	.538^*^	.644^**^	.607^**^	.681^**^	.145	.214
	p =	.378	**.026**	**.005**	**.010**	**.003**	.579	.410
*EAP1*	Rho=	.385	1.000	.544^*^	.490^*^	.860^**^	.537^*^	.279
	p =	.127		**.024**	**.046**	**.000**	**.026**	.277
*HWP1*	Rho=	.868^**^	.338	.821^**^	.571^*^	.532^*^	.615^**^	.201
	p =	**.000**	.184	**.000**	**.017**	**.028**	**.009**	.439
*MNN4*	Rho=	.350	.554^*^	.645^**^	.537^*^	.672^**^	.493^*^	.309
	p =	.168	**.021**	**.005**	**.026**	**.003**	**.045**	.228
*PMR1*	Rho=	.333	.635^**^	.527^*^	.534^*^	.784^**^	.233	.377
	p =	.191	**.006**	**.030**	**.027**	**.000**	.368	.135
*BCR1*	Rho=	.262	.250	.539^*^	.566^*^	.485^*^	.306	.522^*^
	p =	.309	.333	**.026**	**.018**	**.048**	.232	**.032**
*CPH1*	Rho=	.708^**^	.544^*^	1.000	.833^**^	.748^**^	.547^*^	.321
	p =	**.001**	**.024**		**.000**	**.001**	**.023**	.209
*EFG1*	Rho=	.257	.520^*^	.576^*^	.645^**^	.605^*^	.409	.392
	p =	.319	**.033**	**.016**	**.005**	**.010**	.103	.119
*TUP1*	Rho=	.713^**^	.397	.397	.463	.544^*^	.684^**^	.444
	p =	**.001**	.115	.115	.061	**.024**	**.002**	.074
*SAP1*	Rho=	.356	.523^*^	.794^**^	.744^**^	.730^**^	.251	.413
	p =	.160	**.031**	**.000**	**.001**	**.001**	.332	.100
*SAP2*	Rho=	.091	.554^*^	.478	.417	.654^**^	.172	.292
	p =	.729	**.021**	.052	.096	**.004**	.510	.256
*SAP5*	Rho=	.544^*^	.490^*^	.833^**^	1.000	.676^**^	.395	.539^*^
	p =	**.024**	.046	.000		**.003**	.117	**.026**
*SAP6*	Rho=	.371	.772^**^	.637^**^	.627^**^	.869^**^	.320	.284
	p =	.143	**.000**	**.006**	**.007**	**.000**	.211	.269
*PLB1*	Rho=	.593^*^	.860^**^	.748^**^	.676^**^	1.000	.532^*^	.434
	p =	**.012**	**.000**	**.001**	**.003**		**.028**	.082
*CDR1*	Rho=	.779^**^	.537^*^	.547^*^	.395	.532^*^	1.000	.262
	p =	**.000**	**.026**	**.023**	.117	**.028**		.309
*ZAP1*	Rho=	.365	.279	.321	.539^*^	.434	.262	1.000
	p =	.149	.277	.209	**.026**	.082	.309	

*Correlation is significant at the 0.05 level (2-tailed), ** Correlation is significant at the 0.01 level (2-tailed).

a. List wise N = 17.

## Discussion

*C. albicans* is an important pathogen. It is the fourth most common organism isolated from total bloodstream infections [2] and continues to carry a high mortality. The presence of medical devices such as central venous catheters (CVC’s) are known to be important risk factors [14] suggesting that biofilm formation is a key feature in the pathogenesis of candidaemia. The past decade has seen a significant leap in our knowledge and understanding of the biology of *C. albicans* biofilms, particularly with respect to the molecular basis of their development and homeostasis [15]. However, in the clinical setting it is generally assumed that all *C. albicans* isolates have the capacity to form biofilms, but often with little regard to individual differences within the species when managing the infection. Here we demonstrate that *C. albicans* display heterogeneous biofilm characteristics, and these strain differences have important implications with respect to treatment and pathogenicity.

Previous studies have reported very defined categories in their analysis of association with clinical outcomes, i.e. biofilm formers and non-biofilm formers [8,16]. However, these important studies fail to take into account the heterogeneous nature of individual clinical isolates forming biofilms, which based on their metabolic XTT values can range from 0.125 to 1.358 [16]. When looking for clinical correlations with these phenotypes then important information can be missed, as the isolates at either end of the biofilm forming spectrum may lead to different clinical outcomes. We therefore aimed to evaluate and categorise *C. albicans* biofilms into distinct levels of biofilm formation to determine if their biological features were significantly different. Initially we categorised biofilms grown in RPMI using a biomass stain [17] and followed this up with dry weight analysis, which differentiated clinical isolates into defined groupings. This approach was used in preference to metabolic assays due to the highly variable nature of XTT from strain to strain [16]. Moreover, XTT’s mainstay usefulness is limited to antifungal drug testing of biofilms [11,18]. Our classification, based initially on biomass, was supported by observations on a macro- and microscopic level where it was clear that numerous cells consisting of hyphae and yeasts were visible in HBF, whereas scant layers of yeast cells were observed for LBF. We also investigated CSH as an additional biofilm positive feature, as previous studies have also shown a link between biofilm biomass and CSH [13,19]. This study confirms that CSH impacts different phases of biofilm development, which is in agreement with previous work where it was shown that cells dispersed from mature biofilms were more hydrophobic than those dispersed from earlier stages of biofilm development [20]. Furthermore, it has been shown hydrophobic cells are more adherent [21], and therefore it is unsurprising that CSH was increased in HBF isolates. Based on our overall approach to biofilm categorisation we can be confident in the phenotypes selected for further detailed analysis. We do however concede there are caveats to defining levels of biofilm, and this requires further work and collaboration between groups to establish a standardised method.

One of the key defining features of *C. albicans* biofilms is their insensitivity to sterol active antifungal agents [5]. We examined azole treatment, which unsurprisingly demonstrated poor activity overall with no group differences, presumably through adaptive resistance mechanisms as previously described [22-24]. Notably, AMB was less effective against HBF biofilms than LBF, which we hypothesise is due to the inability of the compound to permeate easily throughout the dense physical structure of the cells encased within ECM [25]. We purposely excluded echinocandins from this study as these have been shown to be an effective anti-biofilm antifungals, therefore quantifying differences in activity against the two populations would be difficult [26]. These observations may have implications to whether a patient responds to antifungal therapy, as Tumbarello and colleagues (2012) demonstrated that inadequate antifungal therapy (azoles) and the presence of an indwelling venous catheter were key predictors of patient mortality and hospital length of stay in patients infected with biofilm forming isolates [8]. Guidelines have also suggested that removal of the catheter is an important factor in improving clinical outcomes, again supporting the notion that biofilm formation has a crucial role in clinical outcomes [27,28]. Given the importance of these infections, efficient and appropriate treatment in candidaemia cases has been highlighted [29,30], as failure to treat quickly and effectively has profound consequences on mortality statistics [31].

We decided to test the hypothesis that clinical isolates capable of forming robust biofilms were more pathogenic, which may be a reason for their apparent role in infections with increased mortality [16]. Previous experimental work has shown that cells dispersed from biofilms are more cytotoxic and kill mice quicker than the equivalent planktonic cells [7]. Using a *G. mellonella* model we showed that HBF isolates caused significantly greater mortality rates than LBF isolates, a finding supported elsewhere [32]. In addition, another study investigated the virulence of *C. albicans* isolates with varying levels of biofilm formation and found that mice infected with a LBF had increased survival rates compared to isolates that were infected with HBF [33]. Histological analysis of the infected larvae displayed similar cell morphology of yeast and filamentous hyphae as observed in SEM images of LBF and HBF, respectively. This is in agreement with a previous study that showed filamentation plays a role in killing *G. mellonella* larvae [34].

Filamentous growth is a characteristic feature of *C. albicans* biofilm formation. Defective hyphal formation through deletion of *EFG1* has been shown to lead to low levels of biofilm growth [35]. Given our growing knowledge of key biofilm related genes we decided to investigate transcriptional changes to determine whether these are truly represented amongst clinical isolates, and therefore could be used as a more robust way to categorise biofilm formation and as potential diagnostic targets of HBF isolates. *ACT1,* the stably expressed housekeeping gene, as reported elsewhere [36-38], enabled these relative comparisons. Regulation of biofilm related genes were shown to influence an isolates biomass within clinical isolates, echoing work carried out by other groups [39,40]. Cluster analysis of the selected biofilm related genes showed a good association with functional classes of genes, such as adhesins and proteinases, suggesting that both LBF and HBF had conserved pathways in the basic developmental phases of biofilm growth. However, individual gene expression profiles were inconclusive, showing very few clear independent significant differences, though gene expression proved interesting at 4 h. We investigated the overall biomass at 24 h and examined how 4 h gene expression related to this. Overall *HWP1* was the most highly regulated at this time point in both LBF and HBF, as has been shown elsewhere [39], though no significant differences between the populations were observed. Seven other genes were however shown to have significant positive correlations with biomass. The most significant was *CDR1*, which was unsurprising as it has been shown to be transiently expressed in different biofilm studies, though does not correspond directly to antifungal resistance [41,42]. *PLB1* was showed to be significantly correlated with another 16 genes including biomass, though expression appeared constitutively low level within the biofilm, which is agreement with previous studies [39], and may have an accessory role in the degradation of host tissue alongside *SAP’s*. Of these, *SAP5* was shown to be highly expressed in mature biofilms, and correlated with biomass and 13 other genes. We previously reported that *SAP5* was associated with higher levels of expression in *in vitro* biofilms formed from denture stomatitis *C. albicans* isolates [43]. In addition, Nailis and colleagues demonstrated its crucial role both in a reconstituted human epithelial model and within *in vivo* biofilms [39]. Furthermore, the role of *Sap5* in biofilm formation has recently been demonstrated in BSI, where its expression was significantly increased when compared to planktonic counterparts [44]. Adhesins, such as *ALS3* was also upregulated, which has previously been identified *t*o be involved in biofilm formation, particularly at early stages of biofilm development (0–6 h) [45,46], where *C. albicans* mutants lacking this gene produce sparse biofilms on catheter material *in vitro*[47]. *EAP1*, though showing no clear independent association with biofilm formation *per se*, did show a clear correlation with biomass and 11 other genes. Its importance in biofilm formation has been reported previously [48]. Of interest was the positive correlation with *ZAP1* expression at 4 h, which is a negative regulator of matrix production [49]. It did positively correlate with *BCR1*, the global regulator of biofilm formation, suggesting that the early interaction between their proteins may be important for downstream construction of the biofilm. Collectively the data highlighted the importance of looking at multiple genes at once opposed to single gene targets.

## Conclusions

Overall, we have categorised isolates based on biological properties relating to biofilm characteristics, and evaluated these in models of infection and treatment, where we have shown clear differences in virulence. In an attempt to create a molecular basis of categorising these strains we have used gene expression studies, and showed that individual gene expression analysis of the biofilm related genes to differentiate and categorise biofilm-forming isolates may be futile. Instead, we have shown that taking a defined panel of genes during early biofilm growth may be more informative. In particular, the panel of genes such as *SAP5*, *HWP1*, *EAP1, PLB1* and *CDR1* investigated in tandem could constitute an important step towards diagnostics of *C. albicans* biofilm formation, though the use of transcriptomics, such as RNA-Seq, may prove useful in identifying novel diagnostic targets. Further work is required to determine why some patients succumb to *C. albicans* biofilms whereas others do not, as the HBF isolates do have an increased pathogenic potential and are more difficult to manage with antifungal agents.

## Methods

### Culture conditions and standardisation

*Candida albicans* SC5314, 3153A and a series of routine patient anonymised clinical bloodstream isolates (n = 42) collected under the approval of the NHS Scotland Caldicott Gaurdians from the Royal Hospital for Sick Children (Yorkhill Division), Glasgow, UK, as part of candidaemia epidemiology surveillance study. All clinical isolates obtained during this period were independently identified using Colorex *Candida* chromogenic plates (E&O Laboratories Ltd, Bonnybridge, UK) and were stored in Microbank® vials (Pro-Lab Diagnostics, Cheshire, UK) at −80°C until further use. These isolates were sub-cultured onto Sabouraud’s dextrose agar (SAB [Sigma-Aldrich, Dorset, UK]). Plates were incubated at 30°C for 48 h and maintained at 4°C. Isolates were propagated in yeast peptone dextrose (YPD) medium (Sigma-Aldrich, Dorset, UK), washed by centrifugation and resuspended in the appropriate media (Sigma-Aldrich) to the desired concentration, as described previously [50].

### Characterisation of *Candida albicans* biofilm formation by clinical isolates

All *C. albicans* clinical isolates (n = 42) were standardised to 1 × 10^6^ cells/mL in RPMI-1640 and biofilms grown in flat-bottomed 96 well microtitre plates at 37°C for 24 h and biomass of each isolate assessed using the crystal violet (cv) assay as previously reported [17], and isolates were grouped based on their level of biomass distribution (OD_570nm_ values). Isolates that fell below the 1^st^ quartile (Q_1_) were classed as having low biofilm formation (LBF), strains with a biomass greater than the 3^rd^ quartile (Q_3_) were deemed isolates with high biofilm formation (HBF), and those that lay in between were classified as intermediate biofilm formation (IBF Q_2_). *C. albicans* biomass was further assessed using dry weight measurements. Selected isolates with LBF and HBF were grown as biofilms in 12 well tissue culture plates for 24 h, as previously described, and the resulting biomass homogenised in 1 mL of PBS using a cell scraper (STARLAB, Milton Keynes, UK). This was then passed through a 0.22 μm filter disc (Satorius Stedim) using a vacuum and filters were dried at 40°C overnight before measuring each isolates dry weight. Uninoculated controls were used for background correction.

### Biofilm visualisation

Representative isolates were also grown within 12 well flat-bottomed tissue culture plates (Corning Incorporated, NY, USA) for 24 h before carefully washing with PBS, stained with cv and then digitally imaged (Canon IXUS 220 HS). For scanning electron microscopy (SEM) representative *C. albicans* clinical isolates defined as LBF and HBF were grown directly onto Thermanox™ coverslips (Nunc, Roskilde, Denmark), then processed and analysed as previously described [51].

### Cellular surface hydrophobicity assay

The cell surface hydrophobicity (CSH) was determined for selected *C. albicans* clinical isolates with LBF (n = 10) and HBF (n = 10). CSH was assessed using the microbial adhesion to hydrocarbon test, with a few modifications [52,53]. Isolates were standardised to 1 × 10^6^ cells/mL in RPMI-1640 and grown as biofilms for 4 and 24 h in 75 cm^2^ flasks (Nunc, Rochester, NY) at 37°C. These were then washed with PBS and the resultant biomass scraped off and homogenised in YPD. Cells were standardised (OD_590nm_ 1.0) and cells transferred into a glass tube and overlaid with 1/5^th^ volume of xylene. Contents were vortexed for 1 min and phases separated over 30 min. The aqueous phase was carefully removed and OD_590nm_ measured. The percentage of hydrophobicity was calculated as ([OD_590nm_ before xylene overlay - OD_590nm_ after xylene overlay]/ OD_590nm_ before xylene overlay) × 100%.

### Antifungal susceptibility testing of biofilms

Antifungal testing to determine minimum inhibitory concentration (MIC) of sessile cells was performed using voriconazole (VRZ) and amphotericin B (AMB) (Sigma-Aldrich, Gillingham, UK) on 24 h preformed biofilms, as previously described in flat-bottomed, 96 well microtitre plates [50]. *C. albicans* LBF (n = 10) and HBF (n = 10) were tested in duplicate, on three separate occasions. Sessile minimum inhibitory concentrations (SMICs) were determined at 80% inhibition using an XTT (2,3-bis(2-methoxy-4-nitro-5-sulfo-phenyl)-2H-tetrazolium-5-caboxanilide) metabolic reduction assay [11].

### *Galleria mellonella* pathogenicity assay

The pathogenicity of *C. albicans* isolates pre-defined as LBF (n = 3) and HBF (n = 3) were assessed using the *G. mellonella* killing assay, as described previously [54]. This biological model has been shown previously to be useful in the study of fungal virulence [32,55,56]. Sixth-instar *G. mellonella* larvae (Livefoods Direct Ltd, UK) were stored in the dark and used within 7 days of shipment. Ten random larvae with a bodyweight of between 200 to 300 mg were used for each group. Overnight YPD cultures of each isolate were washed and standardised to 1 × 10^7^ cells/mL in PBS. Larvae were inoculated using a 50-μl Hamilton syringe with 26 g needle by injecting 10 μL aliquots (1 × 10^5^ cells/larva) into the haemocoel, through the hindmost proleg. In addition, mock inoculated larvae pierced on the proleg with a sterile needle and a PBS inoculated control group were also included in each experiment. The infected larvae were placed in sterile petri dishes, incubated at 37°C and the number of dead larvae were scored daily. Larva was considered dead when it displayed no movement in response to touch together with a dark discolouration of the cuticle. Pathogenicity of LBF and HBF was assessed using a Kaplan-Meier plot with percentage survival monitored over 7 days.

### Histology analysis of infected *Galleria mellonella*

The morphology of the larvae infected with two isolates of *C. albicans* LBF and HBF was examined. Larvae were infected with the respective strains as described previously, and after post-infection (24, 48 and 72 h) larvae were fixed by a direct injection of formalin into the haemocoel and by formalin immersion at room temperature for 24 h. Paraffin embedded samples were then transversally sectioned into four-micron thickness using a microtome (microm HM 3335H, Thermoscientific). Sections were then stained with Periodic Acid-Schiff (PAS) to evaluate *C. albicans* infected cells. Whole larvae sections were examined for characterisation and localisation of nodules by light optical microscope visualisation (Leica microscope, model 020–519.502). Two larvae were processed for each isolate, carried out on three separate occasions.

### Transcriptional analysis of biofilm related genes

*C. albicans* clinical isolates exhibiting LBF (n = 10) and HBF (n = 10) were selected for the analysis of genes related to biofilm formation [57]. Biofilms were grown in 24 well flat-bottomed plates for 4 and 24 h at 37°C, as described above. Following incubation, biofilms were washed with PBS, removed and homogenised using a bead beater, and RNA extracted using the TRIzol™ (Life Technologies, Paisley, UK) method as described previously by our group [42]. Total RNA was DNase (Qiagen, Crawley, UK) treated and purified using an RNeasy MinElute clean up kit (Qiagen, Crawley, UK), as per manufacturer’s instructions. RNA was quantified and quality assessed using a NanoDrop spectrophotometer (ND-1000, ThermoScientific, Loughborough, UK). Next, cDNA was synthesised from 200 ng of total RNA using High Capacity RNA to cDNA kit (Life Technologies, Paisley, UK) in a MyCycler PCR machine (Bio-Rad Laboratories, Hertfordshire, UK), following manufacturers instructions.

All primers utilised for this study for quantitative PCR (qPCR) were designed from their sequences obtained from the *Candida* Genome Database (CGD) website (http://www.candidagenome.org). The web-based primer design software program Primer3 (http://primer3.ut.ee/) was used. Primers were checked for specificity to *C. albicans* using the NIH-BLAST for short or exact nucleotide sequences (http://blast.ncbi.nlm.nih.gov/Blast.cgi). PCR amplification efficiencies of all designed primer sets were optimised prior to gene expression analysis, with efficiencies of 90-110% used in this study. Details of the oligonucleotides primers (Eurogentec, Southampton, UK) used in this study are listed in Table 3. 200 ng cDNA was used in a mastermix containing SYBR® GreenER™ (Life Technologies, Paisley, UK), UV-treated RNase-free water and forward/reverse primers (10 μM), following manufacturers’ instructions. Cycle conditions consisted of 2 min at 50°C, 10 min at 95°C and forty cycles of 15 s at 95°C and 60 s at 60°C. Each parameter (LBF n = 10, HBF n = 10 at 4 and 24 h) was analysed in duplicate using MxProP Quantitative PCR machine and MxProP 3000 software (Stratagene, Amsterdam, Netherlands) and controls consisted of reactions in which reverse transcriptase template were absent. Gene expression was calculated using the ΔCt method where the genes of interest were normalised to the housekeeping gene *Act1*.

**Table 3:** ***Candida albicans *** **primers for real time qPCR**

**Gene**	**Sequence (5' - 3')**
***ALS1***	F - TTCTCATGAATCAGCATCCACAA
	R - CAGAATTTTCACCCATACTTGGTTTC
***ALS3***	F - CAACTTGGGTTATTGAAACAAAAACA
	R - AGAAACAGAAACCCAAGAACAACCT
***ALS5***	F - CTGCCGGTTATCGTCCATTTA
	R - ATTGATACTGGTTATTATCTGAGGGAGAAA
***EAP1***	F - ACCACCACCGGGTATACAAA
	R - GCCATCACATTTGGTGACAG
***HWP1***	F - GCTCAACTTATTGCTATCGCTTATTACA
	R - GACCGTCTACCTGTGGGACAGT
***BCR1***	F - ATTGCCACCAATACCTGCTC
	R - GGCTGTCCATGTTGTTGTTG
***CPH1***	F - ACGCAGCCACAAGCTCTACT
	R - GTTGTGTGTGGAGGTTGCAC
***EFG1***	F - CCAGTGGTGGCAGTAATGTG
	R - CAGTGGCAGCCTTGGTATTT
***TUP1***	F - GCTTCAGGTAACCCATTGTTGAT
	R - CTTCGGTTCCCTTTGAGTTTAGG
***OCH1***	F - TCATCCAATGTTGCGTGAAT
	R - TCATGATATCGCCACCTTCA
***PMR1***	F - GAATCCCCGCAGACATTAGA
	R - GGGCCTGTTTTCACCAGTTA
***MNN4***	F - TGAGCAATCGTCAAAACCAG
	R - GGCGGTTGTCATTTGTTGAT
***MNT2***	F - CGTCAAGGTGCCTGAAGAAT
	R - GAGGAGGAGGAGGATTTTGG
***CDR1***	F - GTACTATCCATCAACCATCAGCACTT
	R - GCCGTTCTTCCACCTTTTTGTA
***MDR1***	F - TCAGTCCGATGTCAGAAAATGC
	R - GCAGTGGGAATTTGTAGTATGACAA
***ZAP1***	F - CGACTACAAACCACCAGCTTCATC
	R - CCCCTGTTGCTCATGTTTTGTT
***ACT1***	F - AAGAATTGATTTGGCTGGTAGAGA
	R - TGGCAGAAGATTGAGAAGAAGTTT

### Clustering and heat map analysis

Differential expression of the selected genes from all isolates with LBF (n = 10) and HBF (n = 10) were assessed by clustering and heat map analysis using GenEx software (Exiqon, Vedbaek, Denmark). In brief, percentage expression data was pre-processed for log transformation and mean values calculated (n = 10 for both LBF and HBF) for each gene before heat map production. Each coloured cell in the heat map represents the variable expression of genes in LBF and HBF at 4 and 24 h time points. An increase in gene expression is represented by red and a down-regulation by green. Clustering techniques were used to show genes with similar expression patterns (co-regulated genes) in each set of isolates. The clustering was performed independently by average linkage and Euclidean distances used as a distance measure for both dimensions in the data.

### Statistical analysis

Graph production, data distribution and statistical analysis were performed using GraphPad Prism (version 4; La Jolla, CA, USA). After assessing whether data conformed to a normal distribution data were transformed where necessary and One Way Analysis of Variance (ANOVA) was used to investigate significant differences between independent groups. A Bonferroni post-test was used to determine statistically significant differences between groups. The *G. mellonella* survival curve was analysed using log rank test. Student t-tests were used to measure statistical differences between the two independent groups assessed in gene expression studies. Statistical significance was achieved if *p < 0.05*. IBM SPSS® (version 20) statistical analysis software was used for correlation analysis. Two-tailed Spearman rho correlation coefficient was determined separately for all 4 and 24 h selected genes expression versus 24 h biomass data. Genes that had a significant correlation with biomass were tested for correlations with the other genes as described above.

## Abbreviations

BSI: Bloodstream infections; ICU: Intensive care unit; CVC: Central venous catheter; ECM: Extracellular matrix; AMB: Amphotericin b; LBF: Low biofilm former; HBF: High biofilm former; CSH: Cell surface hydrophobicity; VRZ: Voriconazole; RPMI: Roswell park memorial institute; YPD: Yeast peptone dextrose; FCS: Fetal calf serum; YNB: Yeast nitrogen base; PBS: Phosphate buffered saline; CV: Crystal violet; XTT: 2,3-bis(2-methoxy-4-nitro-5-sulfo-phenyl)-2H-tetrazolium-5-caboxanilide; SEM: Scanning electron microscopy; MIC: Minimum inhibitory concentration; qPCR: Quantitative polymerase chain reaction; OM: Original magnification; FB: Fat body; SC: Subcuticle; PI: Paraintestinal; PT: Paratracheal; SD: Standard deviation.

## Competing interests

None of the authors have any conflict of interest in publishing this work.

## Authors’ contributions

LS and RR participated in the study design, carried out the experimental studies on biofilms, performed statistical analysis and were responsible for the manuscript. DFL participated in study design, assisted with statistical support and helped draft the manuscript. FP, MF, DT and EB participated in study design and undertook the experimental work related to the *G. mellonella* model. BLJ and CW contributed to study design, data analysis and supervised manuscript writing. KS participated in qPCR analysis and manuscript writing. CJN participated in study design, analysis and supervised manuscript writing. GR conceived the study, participated in study design, data analysis and was responsible for writing and submission of the final manuscript. All authors read and approved the manuscript.

## Authors’ information

Infection and Immunity Research Group, Glasgow Dental School, School of Medicine, College of Medical, Veterinary and Life Sciences, University of Glasgow.

## Supplementary Material

Additional file 1: Figure S1 Optimisation of *C. albicans* biofilms. Standardised *C. albicans* SC5314 and 3153A (1×10^6^ cells/mL) were grown in flat-bottomed 96 well microtitre plates at 37°C for 24, 48 and 72 h in RPMI-1640, YPD + 10% FCS, YNB + 100mM glucose and Spider media. Negative controls were also included. Mature biofilms were carefully washed with PBS, air-dried and biomass quantified by staining each biofilm with 0.05% w/v crystal violet solution. The biofilms were washed and 100% ethanol applied to destain each biofilm. The biomass was quantified spectrophotometrically by reading absorbance at 570nm in a microtitre plate reader (FluoStar Omega, BMG Labtech). Three replicates for each isolate were used and carried out on two separate occasions. Data represents mean ± SEM. Significant differences were observed when comparing RPMI-1640 to all other growth media at 24 h (^*§§*^*p<0.005*, ^§§§^*p<0.0001*), 48 h (^*#*^*p<0.005*, ^###^*p<0.0001*) and 72 h (^†^*p<0.05*, ^†††^*p<0.0001*). Significant differences were also found between periods of biofilm development within each growth media (*p<0.05, **p<0.01). Click here for file

Additional file 2: Table S1 Percentage gene expression in *C. albicans* 4 and 24 h biofilms. Click here for file

## References

[B1] Mensa J Pitart C Marco F Treatment of critically ill patients with candidemia Int J Antimicrob Agents 2008 32 Suppl 2 S93 97 19013347 10.1016/S0924-8579(08)70007-4

[B2] Wisplinghoff H Bischoff T Tallent SM Seifert H Wenzel RP Edmond MB Nosocomial bloodstream infections in US hospitals: analysis of 24,179 cases from a prospective nationwide surveillance study Clin Infect Dis 2004 39 3 309 317 15306996 10.1086/421946

[B3] Kojic EM Darouiche RO *Candida* infections of medical devices Clin Microbiol Rev 2004 17 2 255 267 15084500 10.1128/CMR.17.2.255-267.2004 PMC387407

[B4] Lynch AS Robertson GT Bacterial and fungal biofilm infections Annu Rev Med 2008 59 415 428 17937586 10.1146/annurev.med.59.110106.132000

[B5] Ramage G Rajendran R Sherry L Williams C Fungal biofilm resistance Int J Microbiol 2012 2012 528521 22518145 10.1155/2012/528521 PMC3299327

[B6] Taff HT Nett JE Zarnowski R Ross KM Sanchez H Cain MT Hamaker J Mitchell AP Andes DR A *Candida* biofilm-induced pathway for matrix glucan delivery: implications for drug resistance PLoS Pathog 2012 8 8 e1002848 22876186 10.1371/journal.ppat.1002848 PMC3410897

[B7] Uppuluri P Chaturvedi AK Srinivasan A Banerjee M Ramasubramaniam AK Kohler JR Kadosh D Lopez-Ribot JL Dispersion as an important step in the *Candida albicans* biofilm developmental cycle PLoS Pathog 2010 6 3 e1000828 20360962 10.1371/journal.ppat.1000828 PMC2847914

[B8] Tumbarello M Posteraro B Trecarichi EM Fiori B Rossi M Porta R de Gaetano DK La Sorda M Spanu T Fadda G Cauda R Sanguinetti M Biofilm production by *Candida* species and inadequate antifungal therapy as predictors of mortality for patients with candidemia J Clin Microbiol 2007 45 6 1843 1850 17460052 10.1128/JCM.00131-07 PMC1933062

[B9] Daniels KJ Park YN Srikantha T Pujol C Soll DR Impact of environmental conditions on the form and function of *Candida albicans* biofilms Eukaryot Cell 2013 12 10 1389 1402 23954841 10.1128/EC.00127-13 PMC3811334

[B10] Kucharikova S Tournu H Lagrou K Van Dijck P Bujdakova H Detailed comparison of *Candida albicans* and *Candida glabrata* biofilms under different conditions and their susceptibility to caspofungin and anidulafungin J Med Microbiol 2011 60 Pt 9 1261 1269 21566087 10.1099/jmm.0.032037-0

[B11] Pierce CG Uppuluri P Tristan AR Wormley FL Jr Mowat E Ramage G Lopez-Ribot JL A simple and reproducible 96-well plate-based method for the formation of fungal biofilms and its application to antifungal susceptibility testing Nat Protoc 2008 3 9 1494 1500 18772877 10.1038/nport.2008.141 PMC2741160

[B12] Samaranayake YH Cheung BP Parahitiyawa N Seneviratne CJ Yau JY Yeung KW Samaranayake LP Synergistic activity of lysozyme and antifungal agents against *Candida albicans* biofilms on denture acrylic surfaces Arch Oral Biol 2009 54 2 115 126 19038377 10.1016/j.archoralbio.2008.09.015

[B13] Galan-Ladero MA Blanco-Blanco MT Hurtado C Perez-Giraldo C Blanco MT Gomez-Garcia AC Determination of biofilm production by *Candida tropicalis* isolated from hospitalized patients and its relation to cellular surface hydrophobicity, plastic adherence and filamentation ability Yeast 2013 30 331 339 23775541 10.1002/yea.2965

[B14] Gamaletsou MN Walsh TJ Zaoutis T Pagoni M Kotsopoulou M Voulgarelis M Panayiotidis P Vassilakopoulos T Angelopoulou MK Marangos M Spyridonidis A Kofteridis D Pouli A Sotiropoulos D Matsouka P Argyropoulou A Perloretzou S Leckerman K Manaka A Oikonomopoulos P Daikos G Petrikkos G Sipsas NV A prospective, cohort, multicentre study of candidaemia in hospitalized adult patients with haematological malignancies Clin Microbiol Infect 2013 20 050 057 10.1111/1469-0691.12312 23889746

[B15] Blankenship JR Mitchell AP How to build a biofilm: a fungal perspective Curr Opin Microbiol 2006 9 6 588 594 17055772 10.1016/j.mib.2006.10.003

[B16] Tumbarello M Fiori B Trecarichi EM Posteraro P Losito AR De Luca A Sanguinetti M Fadda G Cauda R Posteraro B Risk factors and outcomes of candidemia caused by biofilm-forming isolates in a tertiary care hospital PLoS One 2012 7 3 e33705 22479431 10.1371/journal.pone.0033705 PMC3316499

[B17] Jose A Coco BJ Milligan S Young B Lappin DF Bagg J Murray C Ramage G Reducing the incidence of denture stomatitis: are denture cleansers sufficient? J Prosthodont 2010 19 4 252 257 20113395 10.1111/j.1532-849X.2009.00561.x

[B18] Nett JE Cain MT Crawford K Andes DR Optimizing a *Candida* biofilm microtiter plate model for measurement of antifungal susceptibility by tetrazolium salt assay J Clin Microbiol 2011 49 4 1426 1433 21227984 10.1128/JCM.02273-10 PMC3122839

[B19] Blanco MT Sacristan B Lucio L Blanco J Perez-Giraldo C Gomez-Garcia AC Cell surface hydrophobicity as an indicator of other virulence factors in *Candida albicans* Revista iberoamericana de micologia 2010 27 4 195 199 20849975 10.1016/j.riam.2010.09.001

[B20] Bujdáková H Didiášová M Drahovská H Černáková L Role of cell surface hydrophobicity in *Candida albicans* biofilm Cent Eur J Biol 2013 8 3 259 262

[B21] Perez A Ramage G Blanes R Murgui A Casanova M Martinez JP Some biological features of *Candida albicans* mutants for genes coding fungal proteins containing the CFEM domain FEMS Yeast Res 2011 11 3 273 284 21205162 10.1111/j.1567-1364.2010.00714.x

[B22] Akins RA An update on antifungal targets and mechanisms of resistance in *Candida albicans* Med Mycol 2005 43 4 285 318 16110776 10.1080/13693780500138971

[B23] Cannon RD Lamping E Holmes AR Niimi K Tanabe K Niimi M Monk BC *Candida albicans* drug resistance another way to cope with stress Microbiology 2007 153 Pt 10 3211 3217 17906120 10.1099/mic.0.2007/010405-0

[B24] White TC Increased mRNA levels of ERG16, CDR, and MDR1 correlate with increases in azole resistance in *Candida albicans* isolates from a patient infected with human immunodeficiency virus Antimicrob Agents Chemother 1997 41 7 1482 1487 9210670 10.1128/aac.41.7.1482 PMC163944

[B25] Nett JE Crawford K Marchillo K Andes DR Role of Fks1p and matrix glucan in *Candida albicans* biofilm resistance to an echinocandin, pyrimidine, and polyene Antimicrob Agents Chemother 2010 54 8 3505 3508 20516280 10.1128/AAC.00227-10 PMC2916329

[B26] Bachmann SP VandeWalle K Ramage G Patterson TF Wickes BL Graybill JR Lopez-Ribot JL In vitro activity of caspofungin against *Candida albicans* biofilms Antimicrob Agents Chemother 2002 46 11 3591 3596 12384370 10.1128/AAC.46.11.3591-3596.2002 PMC128731

[B27] Andes DR Safdar N Baddley JW Playford G Reboli AC Rex JH Sobel JD Pappas PG Kullberg BJ Impact of treatment strategy on outcomes in patients with candidemia and other forms of invasive candidiasis: a patient-level quantitative review of randomized trials Clin Infect Dis 2012 54 8 1110 1122 22412055 10.1093/cid/cis021

[B28] Cornely OA Bassetti M Calandra T Garbino J Kullberg BJ Lortholary O Meersseman W Akova M Arendrup MC Arikan-Akdagli S Bille J Castagnola E Cuenca-Estrella M Donnelly JP Groll AH Herbrecht R Hope WW Jensen HE Lass-Florl C Petrikkos G Richardson MD Roilides E Verweij PE Viscoli C Ullmann AJ ESCMID* guideline for the diagnosis and management of *Candida* diseases 2012: non-neutropenic adult patients Clin Microbiol Infect 2012 18 Suppl 7 19 37 23137135 10.1111/1469-0691.12039

[B29] Almirante B Rodriguez D Park BJ Cuenca-Estrella M Planes AM Almela M Mensa J Sanchez F Ayats J Gimenez M Saballs P Fridkin SK Morgan J Rodriguez-Tudela JL Warnock DW Pahissa A Epidemiology and predictors of mortality in cases of *Candida* bloodstream infection: results from population-based surveillance, barcelona, Spain, from 2002 to 2003 J Clin Microbiol 2005 43 4 1829 1835 15815004 10.1128/JCM.43.4.1829-1835.2005 PMC1081396

[B30] Garey KW Rege M Pai MP Mingo DE Suda KJ Turpin RS Bearden DT Time to initiation of fluconazole therapy impacts mortality in patients with candidemia: a multi-institutional study Clin Infect Dis 2006 43 1 25 31 16758414 10.1086/504810

[B31] Kollef M Micek S Hampton N Doherty JA Kumar A Septic shock attributed to *Candida* infection: importance of empiric therapy and source control Clin Infect Dis 2012 54 12 1739 1746 22423135 10.1093/cid/cis305

[B32] Cirasola D Sciota R Vizzini L Ricucci V Morace G Borghi E Experimental biofilm-related Candida infections Future Microbiol 2013 8 6 799 805 23701334 10.2217/fmb.13.36

[B33] Hasan F Xess I Wang X Jain N Fries BC Biofilm formation in clinical *Candida* isolates and its association with virulence Microbes Infect 2009 11 8–9 753 761 19409507 10.1016/j.micinf.2009.04.018 PMC2715444

[B34] Fuchs BB Eby J Nobile CJ El Khoury JB Mitchell AP Mylonakis E Role of filamentation in *Galleria mellonella* killing by *Candida albicans* Microbes Infect 2010 12 6 488 496 20223293 10.1016/j.micinf.2010.03.001 PMC2883670

[B35] Ramage G VandeWalle K Lopez-Ribot JL Wickes BL The filamentation pathway controlled by the Efg1 regulator protein is required for normal biofilm formation and development in *Candida albicans* FEMS Microbiol Lett 2002 214 1 95 100 12204378 10.1111/j.1574-6968.2002.tb11330.x

[B36] Lepak A Nett J Lincoln L Marchillo K Andes D Time course of microbiologic outcome and gene expression in *Candida albicans* during and following in vitro and in vivo exposure to fluconazole Antimicrob Agents Chemother 2006 50 4 1311 1319 16569846 10.1128/AAC.50.4.1311-1319.2006 PMC1426956

[B37] Nailis H Coenye T Van Nieuwerburgh F Deforce D Nelis HJ Development and evaluation of different normalization strategies for gene expression studies in *Candida albicans* biofilms by real-time PCR BMC Mol Biol 2006 7 25 16889665 10.1186/1471-2199-7-25 PMC1557526

[B38] Nett JE Lepak AJ Marchillo K Andes DR Time course global gene expression analysis of an in vivo *Candida* biofilm J Infect Dis 2009 200 2 307 313 19527170 10.1086/599838 PMC3159582

[B39] Nailis H Kucharikova S Ricicova M Van Dijck P Deforce D Nelis H Coenye T Real-time PCR expression profiling of genes encoding potential virulence factors in *Candida albicans* biofilms: identification of model-dependent and -independent gene expression BMC Microbiol 2010 10 114 20398368 10.1186/1471-2180-10-114 PMC2862034

[B40] Uppuluri P Dinakaran H Thomas DP Chaturvedi AK Lopez-Ribot JL Characteristics of *Candida albicans* biofilms grown in a synthetic urine medium J Clin Microbiol 2009 47 12 4078 4083 19794044 10.1128/JCM.01377-09 PMC2786631

[B41] Mukherjee PK Chandra J Kuhn DM Ghannoum MA Mechanism of fluconazole resistance in *Candida albicans* biofilms: phase-specific role of efflux pumps and membrane sterols Infect Immun 2003 71 8 4333 4340 12874310 10.1128/IAI.71.8.4333-4340.2003 PMC165995

[B42] Ramage G Bachmann S Patterson TF Wickes BL Lopez-Ribot JL Investigation of multidrug efflux pumps in relation to fluconazole resistance in *Candida albicans* biofilms J Antimicrob Chemother 2002 49 6 973 980 12039889 10.1093/jac/dkf049

[B43] Ramage G Coco B Sherry L Bagg J Lappin DF In vitro Candida albicans biofilm induced proteinase activity and SAP8 expression correlates with in vivo denture stomatitis severity Mycopathologia 2012 174 1 11 19 22302440 10.1007/s11046-012-9522-2

[B44] Joo MY Shin JH Jang HC Song ES Kee SJ Shin MG Suh SP Ryang DW Expression of SAP5 and SAP9 in *Candida albicans* biofilms: comparison of bloodstream isolates with isolates from other sources Med Mycol 2013 51 8 892 896 23971863 10.3109/13693786.2013.824623

[B45] Nailis H Vandenbroucke R Tilleman K Deforce D Nelis H Coenye T Monitoring ALS1 and ALS3 gene expression during in vitro *Candida albicans* biofilm formation under continuous flow conditions Mycopathologia 2009 167 1 9 17 18683080 10.1007/s11046-008-9148-6

[B46] Zhao X Daniels KJ Oh SH Green CB Yeater KM Soll DR Hoyer LL *Candida albicans* Als3p is required for wild-type biofilm formation on silicone elastomer surfaces Microbiology 2006 152 Pt 8 2287 2299 16849795 10.1099/mic.0.28959-0 PMC2583121

[B47] Liu Y Filler SG *Candida albicans* Als3, a multifunctional adhesin and invasin Eukaryot Cell 2011 10 2 168 173 21115738 10.1128/EC.00279-10 PMC3067396

[B48] Li F Svarovsky MJ Karlsson AJ Wagner JP Marchillo K Oshel P Andes D Palecek SP Eap1p, an adhesin that mediates *Candida albicans* biofilm formation in vitro and in vivo Eukaryot Cell 2007 6 6 931 939 17416898 10.1128/EC.00049-07 PMC1951519

[B49] Nobile CJ Nett JE Hernday AD Homann OR Deneault JS Nantel A Andes DR Johnson AD Mitchell AP Biofilm matrix regulation by *Candida albicans* Zap1 PLoS Biol 2009 7 6 e1000133 19529758 10.1371/journal.pbio.1000133 PMC2688839

[B50] Ramage G Vande Walle K Wickes BL Lopez-Ribot JL Standardized method for in vitro antifungal susceptibility testing of *Candida albicans* biofilms Antimicrob Agents Chemother 2001 45 9 2475 2479 11502517 10.1128/AAC.45.9.2475-2479.2001 PMC90680

[B51] Erlandsen SL Kristich CJ Dunny GM Wells CL High-resolution visualization of the microbial glycocalyx with low-voltage scanning electron microscopy: dependence on cationic dyes J Histochem Cytochem 2004 52 11 1427 1435 15505337 10.1369/jhc.4A6428.2004 PMC3957825

[B52] Yoshijima Y Murakami K Kayama S Liu D Hirota K Ichikawa T Miyake Y Effect of substrate surface hydrophobicity on the adherence of yeast and hyphal *Candida* Mycoses 2010 53 3 221 226 19671080 10.1111/j.1439-0507.2009.01694.x

[B53] Rosenberg M Gutnick D Rosenberg E Adherence of bacteria to hydrocarbons: a simple method for measuring cell-surface hydrophobicity Med Microbiol 1980 9 29 33

[B54] Cotter G Doyle S Kavanagh K Development of an insect model for the in vivo pathogenicity testing of yeasts FEMS immunology and medical microbiology 2000 27 2 163 169 10640612 10.1111/j.1574-695X.2000.tb01427.x

[B55] Gago S Garcia-Rodas R Cuesta I Mellado E Alastruey-Izquierdo A *Candida parapsilosis, Candida orthopsilosis*, and *Candida metapsilosis* virulence in the non-conventional host *Galleria mellonella* Virulence 2014 5 2 278 285 24193303 10.4161/viru.26973 PMC3956503

[B56] Fallon J Kelly J Kavanagh K Galleria mellonella as a model for fungal pathogenicity testing Methods Mol Biol 2012 845 469 485 22328396 10.1007/978-1-61779-539-8_33

[B57] Finkel JS Mitchell AP Genetic control of *Candida albicans* biofilm development Nat Rev Microbiol 2011 9 2 109 118 21189476 10.1038/nrmicro2475 PMC3891587

