# Supplementary figures and images for: Biofilms formed by Candida albicans bloodstream isolates display phenotypic and transcriptional heterogeneity that are associated with resistance and pathogenicity
Source: BMC Microbiol. 2014 Jul 5;14:182. doi: 10.1186/1471-2180-14-182 (PMC4105547; doi:10.1186/1471-2180-14-182)

## Slide 1
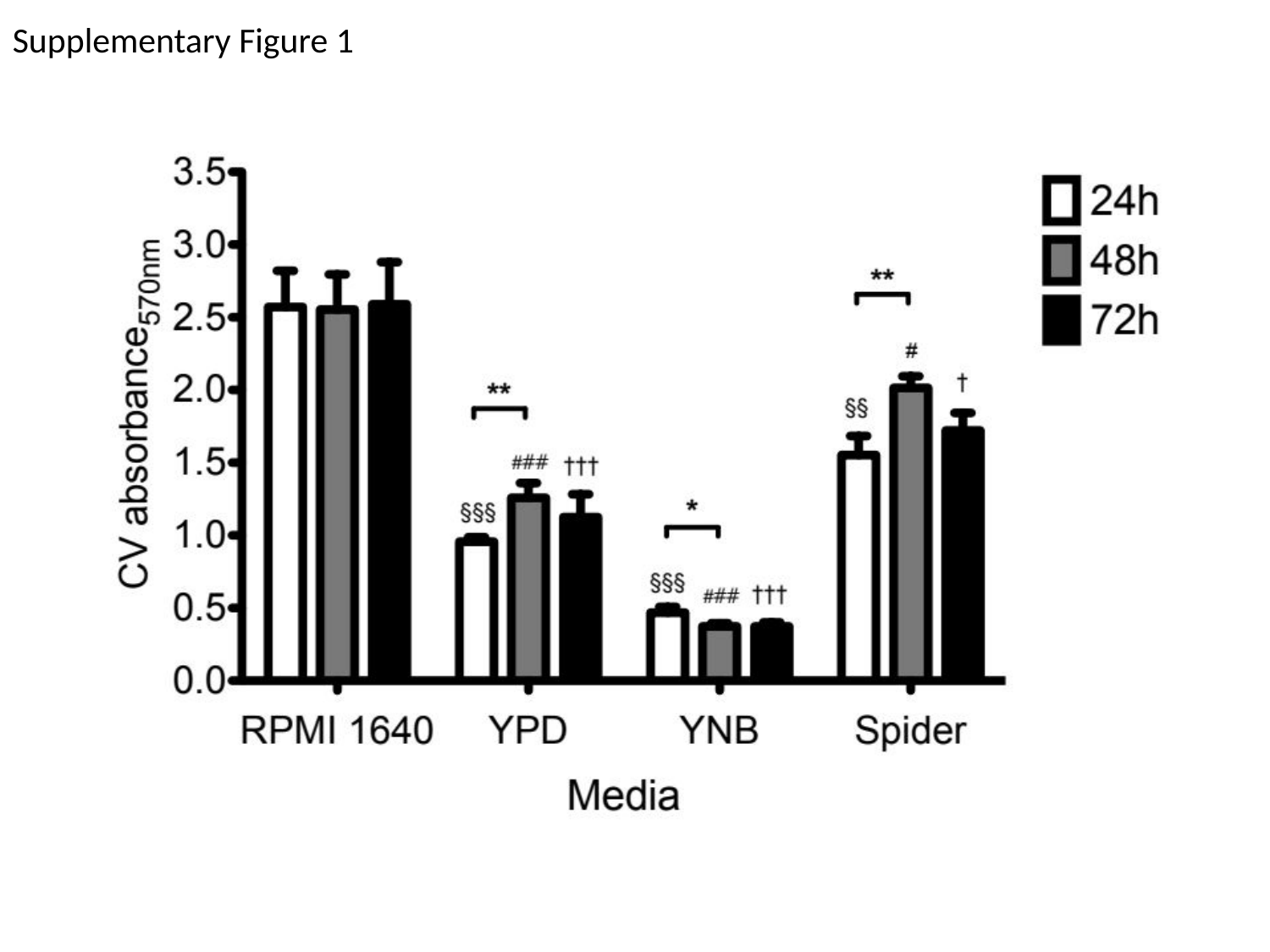

Supplementary Figure 1

Supplement: Additional file 1: Figure S1 — Optimisation of C. albicans biofilms. Standardised C. albicans SC5314 and 3153A (1×10^6^ cells/mL) were grown in flat-bottomed 96 well microtitre plates at 37°C for 24, 48 and 72 h in RPMI-1640, YPD + 10% FCS, YNB + 100mM glucose and Spider media. Negative controls were also included. Mature biofilms were carefully washed with PBS, air-dried and biomass quantified by staining each biofilm with 0.05% w/v crystal violet solution. The biofilms were washed and 100% ethanol applied to destain each biofilm. The biomass was quantified spectrophotometrically by reading absorbance at 570nm in a microtitre plate reader (FluoStar Omega, BMG Labtech). Three replicates for each isolate were used and carried out on two separate occasions. Data represents mean ± SEM. Significant differences were observed when comparing RPMI-1640 to all other growth media at 24 h ( §§ p<0.005, §§§p<0.0001), 48 h ( # p<0.005, ###p<0.0001) and 72 h (†p<0.05, †††p<0.0001). Significant differences were also found between periods of biofilm development within each growth media (*p<0.05, **p<0.01). [file 1471-2180-14-182-S1.pptx]
