# Supplementary material for: Biofilms formed by Candida albicans bloodstream isolates display phenotypic and transcriptional heterogeneity that are associated with resistance and pathogenicity
Source: BMC Microbiol. 2014 Jul 5;14:182. doi: 10.1186/1471-2180-14-182 (PMC4105547; doi:10.1186/1471-2180-14-182)
Supplement: Additional file 2: Table S1 — Percentage gene expression in C. albicans 4 and 24 h biofilms. [file 1471-2180-14-182-S2.doc]

Supplementary Table 1: Percentage gene expression in *C. albicans* 4 and 24 h biofilms

|  | | **4 h biofilm** | | | | **24 h biofilm** | | | |
| --- | --- | --- | --- | --- | --- | --- | --- | --- | --- |
|  |  | **LBF** | | **HBF** | | **LBF** | | **HBF** | |
| **Function** | **Gene** | Mean % expression | ± SD | Mean % expression | ± SD | Mean % expression | ± SD | Mean % expression | ± SD |
| **Adhesion** | *ALS1* | 22.64 | 14.40 | 28.94 | 40.54 | 69.50 | 58.04 | 76.14 | 44.71 |
|  | *ALS3* | 58.71 | 43.94 | 82.59 | 42.00 | 27.05 | 21.21 | 69.95 | 64.06 |
|  | *ALS5* | 1.287 | 1.128 | 1.959 | 2.831 | 6.694 | 4.206 | 11.04 | 9.778 |
|  | *EAP1* | 1.093 | 0.7115 | 2.154 | 1.285 | 11.91 | 5.52 | 9.345 | 6.155 |
|  | *HWP1* | 216.30 | 147.10 | 179.80 | 46.09 | 36.45 | 18.38 | 190.60 | 144.70 |
| **Glycosylated mannoproteins** | *OCH1* | 0.6632 | 0.9831 | 1.231 | 2.336 | 2.207 | 1.484 | 2.207 | 1.484 |
|  | *PMR1* | 6.823 | 3.743 | 7.662 | 5.055 | 16.56 | 3.87 | 15.20 | 5.35 |
|  | *MNN4* | 7.096 | 4.081 | 1.299 | 1.839 | 45.13 | 23.89 | 75.18 | 40.34 |
|  | *MNT2* | 2.524 | 2.366 | 10.57 | 11.66 | 37.04 | 13.88 | 45.87 | 12.04 |
| **Transcription factors** | *CPH1* | 3.461 | 1.954 | 5.252 | 2.169 | 8.344 | 2.277 | 7.82 | 3.039 |
|  | *BCR1* | 5.46 | 2.179 | 3.163 | 2.179 | 49.10 | 23.08 | 40.17 | 13.74 |
|  | *EFG1* | 43.28 | 16.94 | 29.52 | 15.19 | 33.50 | 12.94 | 24.12 | 17.56 |
|  | *TUP1* | 23.6 | 17.51 | 17.34 | 4.93 | 261.80 | 100.00 | 352.70 | 102.20 |
| **Resistance** | *CDR1* | 3.255 | 5.454 | 14.87 | 6.621 | 9.772 | 9.371 | 260.00 | 144.10 |
|  | *MDR1* | 0.1923 | 0.1692 | 0.3027 | 0.7259 | 11.43 | 9.50 | 7.957 | 6.641 |
|  | *ZAP1* | 4.802 | 2.769 | 5.085 | 6.125 | 34.36 | 13.84 | 39.05 | 12.77 |
